# Orthovoltage intraoperative radiation therapy for pancreatic adenocarcinoma

**DOI:** 10.1186/1748-717X-5-105

**Published:** 2010-11-08

**Authors:** Pavan Bachireddy, Diane Tseng, Melissa Horoschak, Daniel T Chang, Albert C Koong, Daniel S Kapp, Phuoc T Tran

**Affiliations:** 1 Department of Radiation Oncology, Stanford Cancer Center, Stanford University, Stanford, CA, USA; 2 Department of Radiation Oncology and Molecular Radiation Sciences, the Sidney Kimmel Comprehensive Cancer Center, Johns Hopkins University, Baltimore, MD, USA

## Abstract

**Purpose:**

To analyze the outcomes of patients from a single institution treated with surgery and orthovoltage intraoperative radiotherapy (IORT) for pancreatic adenocarcinoma.

**Methods:**

We retrospectively reviewed 23 consecutive patients from 1990-2001 treated with IORT to 23 discrete sites with median and mean follow up of 6.5 and 21 months, respectively. Most tumors were located in the head of the pancreas (83%) and sites irradiated included: tumor bed (57%), vessels (26%), both the tumor bed/vessels (13%) and other (4%). The majority of patients (83%) had IORT at the time of their definitive surgery. Three patients had preoperative chemoradiation (13%). Orthovoltage X-rays (200-250 kVp) were employed via individually sized and beveled cone applicators. Additional mean clinical characteristics include: age 64 (range 41-81); tumor size 4 cm (range 1.4-11); and IORT dose 1106 cGy (range 600-1500). Post-operative external beam radiation (EBRT) or chemotherapy was given to 65% and 76% of the assessable patients, respectively. Outcomes measured were infield control (IFC), loco-regional control (LRC), distant metastasis free survival (DMFS), overall survival (OS) and treatment-related complications.

**Results:**

Kaplan-Meier (KM) 2-year IFC, LRC, DMFS and OS probabilities for the whole group were 83%, 61%, 26%, and 27%, respectively. Our cohort had three grade 3-5 complications associated with treatment (surgery and IORT).

**Conclusions:**

Orthovoltage IORT following tumor reductive surgery is reasonably well tolerated and seems to confer in-field control in carefully selected patients. However, distant metastases remain the major problem for patients with pancreatic adenocarcinoma.

## Background

Pancreatic cancer is the fourth leading cause of cancer mortality in the United States, afflicting more than 32,000 people annually. A 5-year survival rate less than 5% highlights the dismal prognosis accompanying this disease [1]. The majority of patients present with locally advanced or metastatic disease, precluding complete surgical resection. Even the minority of patients able to achieve complete surgical resection can only expect a 5-year survival rate of approximately 20% [2,3]. The natural history of resected pancreatic adenocarcinoma has led to introduction of the use of adjuvant chemoradiotherapy regimens following surgery [4,5]. Initial randomized trials demonstrated a survival benefit with adjuvant chemoradiation compared to observation alone, but subsequent trials have suggested either potential harm when compared to adjuvant chemotherapy or lack of benefit [6-10]. However, these negative trials have been criticized for serious flaws in design and quality control [4,11-13]. Issues with tolerance and completion of chemoradiation regimens as well as lack of efficacy of low doses of adjuvant radiation therapy indicate the need to investigate dose escalation [14].

Intraoperative radiation therapy (IORT) is a specialized modality that offers the precise delivery of a high dose of ionizing radiation targeted to the tumor bed and regions at risk to eliminate microscopic disease *in situ *while simultaneously allowing for the displacement and protection of surrounding normal tissue from the treatment. As a result, IORT is well suited as a technique to dose escalate in an attempt to increase local control from radiation therapy. Recent studies suggest that IORT may have a positive impact on local control and possibly even overall survival in patients with localized pancreatic cancer [15-18]. The objectives of this study are to review our experience in patients treated with surgery and orthovoltage IORT for pancreatic adenocarcinoma.

## Methods

We conducted an institutional review board-approved retrospective review of consecutive patients treated between August 1993 and August 2002 with IORT and surgery for pancreatic adenocarcinoma by the Department of Radiation Oncology, Stanford University Medical Center, Stanford, CA. Our cohort consisted of 23 patients selected for IORT on the basis of tumor resectability as well as high likelihood of local-regional recurrence.

Pretreatment evaluation included patient history; complete physical examination; routine laboratory studies; and imaging by computed tomography scan of the abdomen. Informed consent was obtained from all patients before treatment.

Hospital medical records, clinic charts, and radiation oncology records were reviewed. We updated follow-up information in all patients within 1 month before the present study by using examination, data from the referring physician, or direct correspondence with patients or relatives. Follow-up for surviving patients was determined from the day of IORT.

### Treatment of Patients

The majority of patients (83%) had IORT at the time of their definitive surgery. Three patients had preoperative chemoradiation (13%). Surgery was carried out in a dedicated operative suite containing a Philips RT-250 IORT radiation unit (Philips Medical Systems, Best, and The Netherlands). Treatment was delivered with 200-250-kVp orthovoltage X-rays directly over the tumor bed and/or regions at risk including vessels via individually sized and beveled cone applicators. Choice of half-value layer (HVL) filters, 0.57-2.45 mm copper, was based on consideration of dose rate (50-100 cGy/min), residual tumor thickness and underlying tissues. For example, when there is normal bone in the exit of the beam, higher HVL beam (with higher kvp) was used to minimize excess bone dose due to the contribution from the photoelectric effect. Treatment fields were designed to encompass a 0.5- to 1-cm margin around the tumor bed. IORT was administered employing a series of specially designed nickel plated brass circular cones with diameters ranging from 2.5-12.5 cm and bevels of 0°, 15°, 30°, and 45°. The mean IORT dose was 1106 cGy (range 600-1500). All doses were prescribed to the surface, and no bolus was used. Before administration of IORT, maximal efforts were made to mobilize and pack uninvolved small and large intestines and major uninvolved nerves or vessels out of the proposed radiation field. If this was not possible, customized lead shielding was employed to prevent overdosing of vital structures. IORT dose to normal bowel and major nerves was limited to ≤ 12.5 Gy whenever possible. Post-operative external beam radiation (EBRT) or chemotherapy was given to 65% and 75% of the assessable patients, respectively. Post-operative EBRT total dose ranged from 4500-5400 cGy given over 2-3 months. Chemotherapy consisted of concurrent infusion of 5-fluorouracil (5-FU) with one patient receiving both 5-FU and gemcitabine.

### Follow-up

After completion of treatment, patients were evaluated at 1, and 3- to 6-month intervals for disease status and treatment-related complications. Routine evaluation included physical examination, hematology and chemistry profiles. In general chest radiography and abdominal CT were performed every 6 months.

Outcomes analyzed were infield control (IFC), loco-regional control (LRC), distant metastasis free survival (DMFS), overall survival (OS) and treatment-related complications. Intervals were defined from day of IORT to last follow-up or first reported site of failure or death from cancer. Disease relapse in the IORT field was defined as an infield failure, whereas relapse within the compartment of IORT was defined as locoregional failure. The DMFS was defined as survival without distant recurrence (outside the locoregional compartment), and other events were censored. Similarly, OS was scored as death from pancreatic cancer or, if information was lacking, death likely from pancreatic cancer; other competing causes of death were censored.

Margin status was confirmed by pathology report of frozen/permanent sections, and if not available or noninformative, IORT and/or surgical reports were used. Microscopic residual disease was defined as the presence of tumor cells at the surface of the resection margin (0 mm definition), and thus greater than 0 mm margin was considered negative [19]. Patients were also grouped into R0 (gross complete resection with negative margins), R1 (gross complete resection with positive margins), and R2 (macroscopic residual disease) resections.

Complications were scored according to National Cancer Institute Common Terminology Criteria for Adverse Events, version 3.0. The interval for Grade 3-5 (G3-5) complications (complication-free survival [CFS]) CFS was defined as day of IORT to first reported G3-5 complication.

### Statistics

Survival graphs were generated by the product limit method of Kaplan and Meier and log-rank analysis was utilized for differences between proportions. Analysis was facilitated using Prism, version 4.0, by GraphPad (San Diego, CA).

## Results

### Patient Characteristics

Our cohort of 23 patients treated with IORT at 23 sites included 12 women and 11 men. Racial distribution included 15 Caucasians (65%), 4 Asians (17%), 1 African-American (4%), 1 Tongan (4%), 1 Native American (4%), and 1 not otherwise specified (4%). The majority, 18 (78%), endorsed a history of smoking; only 2 (8%) reported extensive alcohol use. Primary sites of disease and original stages for patients in our series were 14 patients with involvement of the pancreatic head only, 10 Stage II, 3 Stage III, 1 Stage IV and one not otherwise specified; two involving head and uncinate process, 1 Stage II and 1 Stage III; one involving head and body, Stage III; one involving head and tail, Stage II; 2 involving body alone, 1 Stage 1 and 1 Stage II; one involving body and tail, Stage 1; one involving tail alone, Stage III; and one arising at the ampulla of Vater. Two patients had failed prior surgeries (8%), one (4%) of which had had prior chemoradiation therapy. Preoperative CA19-9 was obtained on 14 (61%) patients, ranging from 1-2020 (median 123, mean 477). Additional pre-IORT characteristics are listed in Table 1.

**Table 1:** Patient characteristics prior to IORT (n = 23)

Age, mean, years (range)		65 (41-81)
Prior surgery, n (%)		2 (9)
Prior radiotherapy, n (%)		3 (13)
Prior systemic therapy, n (%)		4 (17)
Tumor size, mean, cm (range)		4.1 (1.4-11)
Primary site, n (%):	Head of pancreas	14 (61)
	Head and uncinate/body/tail	4 (17)
	Other*	5 (22)

* - Ampulla of Vater (1), body (2), body and tail (1), tail (1).

### Surgery, IORT, and post-IORT treatment

Whipple procedure (pancreaticoduodenectomy) was performed on 17 tumor sites (74%) and of these, 10 (43%) were felt to have positive gross margins intraoperatively (for summary see Table 2). Margin status showed that 11 sites (all 10 sites with positive gross margins and 1 with a negative gross margin) had microscopic residual disease. Most tumors were located in the head of the pancreas (83%) and sites irradiated included: tumor bed (57%), vessels (26%), both the tumor bed/vessels (13%) and other (4%). IORT was administered with a mean dose of 11.1 Gy and median cone size of 6.25 cm.

**Table 2:** Treatment characteristics

Type of surgery, n (%):	Pancreaticoduodenectomy (Whipple)	17 (74)
	Distal pancreatectomy	2 (9)
	Other *	4 (17)
Resection status, n (%):	R2	8 (35)
	R1	6 (26)
	R0	9 (39)
IORT cone size, median cm (range)		6.25 (5-10)
	R0-R2, mean (range)	11.1 (6-15)
IORT dose, Gy	R2, mean (range)	12.1 (6-15)
	R1, mean (range)	11 (8-12)
	R0, mean (range)	10.3 (8-12)
Post-IORT Treatment:	External beam radiotherapy, n (%)	11 (65)
	XRT dose, mean, Gy (range)	49 (45-54)
	Systemic therapy**, n (%)	13 (76)

* - resection of recurrence (1), surgical bypass (roux-en-y) (2), cholecystecomy/gastrojejunostomy/choledochojejunostomy (1).

** - 5-fluorouracil +/- gemcitabine

Post-operative external beam radiation (EBRT) or chemotherapy was given to 65% and 76% of the assessable patients, respectively. The mean EBRT dose was 48.8 Gy, ranging from 45-54 Gy. Of the assessable patients who did not receive post-operative EBRT, 3 (18%) had received prior EBRT (either neoadjuvant or subsequent to prior surgery), two (12%) had either locally advanced or metastatic disease discovered after surgery, and one (6%) died from post-operative complications (intra-abdominal hemorrhage). Two patients that had neoadjuvant chemoradiation (8%) resulted in measurable responses.

### Outcomes analysis and complications

**Figure 1:**
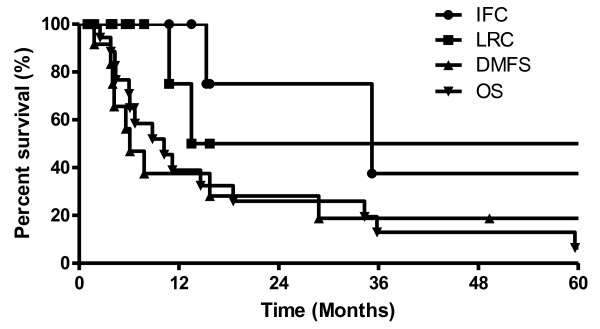
**Outcomes of patients treated with orthovoltage IORT**. Kaplan-Meier (KM) plot of the entire cohort for infield control (IFC), loco-regional control (LRC), distant metastasis free survival (DMFS) and overall survival (OS). Twenty-three patients were treated with surgery and IORT.

Median and mean follow-up of patients was 6.5 and 21 months, respectively. At the time of analysis, 1 out of 16 assessable patients (6%) was alive, and 1 (6%) died from postoperative complications. Kaplan-Meier (KM) 2-year IFC, LRC, DMFS and OS probabilities for the whole group were 83%, 61%, 26%, and 27%, respectively (Figure 1). Our cohort had three G3-5 complications associated with treatment (surgery and IORT) translating into a 2-yr KM G3-5 complication free survival (CFS) of 68%. The G3-5 complications included myocardial infarction [post-operative day (POD) 6], death from intraabdominal hemorrhage (POD 0), neutropenic fever and sepsis (two months post-operative).

## Discussion

We present our analysis of a consecutive series of patients with pancreatic adenocarcinoma treated with surgery and orthovoltage IORT. The 2-year LRC (61%) and 2-year OS (27%) in our series of patients are consistent with previously published series for this historically poor prognostic group of patients [10,16]. We have found that surgery combined with IORT followed by adjuvant chemoradiation seems to improve local control of the tumor, although this may not have necessarily translated into a survival benefit.

The largest single center retrospective cohort study of 203 pancreatic adenocarcinoma patients examined the clinical effectiveness of IORT after surgical resection and found that there was a significant survival benefit in addition to improved local control rates in localized pancreatic cancer [15]; however, the improvement in survival has not been consistent among other studies and may be applicable only to patients with localized disease. In other retrospective studies, it appears that addition of IORT may provide a slight overall survival benefit of 1-2 months in the subset of patients with localized pancreatic cancer [17]. The survival benefit and impact of local control of IORT in pancreatic adenocarcioma has been addressed in an older prospective randomized clinical trial, but the sample size was likely too small to adequately address the question of benefit [20]. The only other prospective clinical trial published involving IORT for pancreatic cancer was designed to test the efficacy of a radiosensitizing drug; even then, the question in the study was not focused on the clinical benefit of IORT [21]. While it is difficult to compare between retrospective series, our data appear consistent with the other reported studies [17].

Residual disease following pancreatic cancer surgery is an important prognostic factor [2,3]. The benefit of IORT is likely from improved local control particularly on minimal-microscopic disease that remains following resection. We did not find residual disease status, R0, R1 or R2, to be an important determinant of IFC, LRC or OS in our study (data not shown). This may reflect the already highly selected population in our series, small cohort size and/or cohort heterogeneity.

Intraoperative orthovoltage units are less commonly used than either mobile or non-mobile linear accelerators, which deliver electron beams [22]. The use of intraoperative orthovoltage RT has been previously described and the dosimetric properties of a unit similar to the one used in our study characterized [23,24]. While the steepness of the fall-off of dose with depth is slower than that for electron beams as reviewed previously [25], our unit provides flexible access to localizations deep in the abdomen, which would be more difficult to treat intraoperatively with linear accelerators. Moreover, the initial costs, shielding requirements, and maintenance of orthovoltage units are less than those of linear accelerators.

The limitations of our retrospective series of IORT for pancreatic adenocarcinoma are inherent in all retrospective clinical study designs. These include, but are not limited to, selection bias, recall bias, heterogeneity of patients and a small number of patients. In addition, the 10-year time span over which our cohort was collected might have allowed for confounding factors associated with improvements with diagnosis, surgical technique, and treatment. Our study of post-IORT outcomes may include a biased patient population with even more advanced disease who are not typical candidates for surgery because of Stanford's surgical expertise and experience with pancreaticoduodenectomies. In our series, 7/23 (30%) of our patients had either stage III or stage IV disease and 11/23 (48%) had gross and/or microscopic residual disease following pancreaticoduodenectomy. However, if anything, this would have biased our results toward poorer outcomes compared to other studies. Because retrospective data is hypothesis generating at best, we fully endorse efforts toward larger randomized clinical trials using IORT, such as the efforts of the International Society of Intraoperative Radiotherapy (see http://www.isiort.org)

In our cohort, we observed a 2-yr KM G3-5 complication free survival of 68%. The complications included myocardial infarction, death from intraabdominal hemorrhage, neutropenic fever, and sepsis. The intraabdominal hemorrhage was unlikely to be from the IORT as the source of the bleeding was clearly outside of the treatment field. These other peri-operative complications are not likely from IORT alone, however, it is not clear whether or not these complications are a result of the surgery itself or if there is a possible contribution of IORT to these complications. Pancreaticoduodenectomies by themselves are large operations that place dramatic stress on the cardiovascular system, predispose the patient to infection, and place them at risk for intraabdominal bleed due to the rich vasculature at the surgical site. In most of the previous studies of IORT for pancreatic adenocarcinoma, IORT does not increase the risks associated with surgery [26-28]. In addition a recent multi-institutional case series, which had the largest sample of patients treated with IORT to date, observed a low frequency of adverse late events after IORT [29] (3%). These were late grade 3-4 gastrointestinal events comprised of colitis, ileus and bleeding.

Finally, for the benefits in local control rates to translate into improvements in survival will require further improvements in systemic therapy. Retrospective studies supporting this notion have recently been published [29-31].

The addition of IORT to cytoreductive surgery requires the balance of the potential for improved local control and survival enhancement with the risk for added toxicity. IORT for pancreatic adenocarcinoma has the potential to improve LRC and clinical outcomes for patients with advanced disease and residual local tumor following surgery.

## Conclusions

Orthovoltage IORT following tumor reductive surgery is reasonably well tolerated and seems to confer in-field control in carefully selected patients. Distant metastases remain the major problem for patients with pancreatic adenocarcinoma.

## Conflicts of interest Notification

The authors declare that they have no competing interests.

## Authors' contributions

PB and DT carried out the clinical review required in the study, analyzed the data and drafted the manuscript. MH helped to carry out the clinical review required in the study. DC and AK participated in the review of the drafted manuscript. DSK and PT conceived of the study, participated in its design, performed the analysis and coordinated and helped to draft the manuscript. All authors read and approved the final manuscript.
